# Conceptualization and validation of an open-source closed-loop deep brain stimulation system in rat

**DOI:** 10.1038/srep09921

**Published:** 2015-04-21

**Authors:** Hemmings Wu, Hartwin Ghekiere, Dorien Beeckmans, Tim Tambuyzer, Kris van Kuyck, Jean-Marie Aerts, Bart Nuttin

**Affiliations:** 1 Research Group Experimental Neurosurgery and Neuroanatomy, KU Leuven, Leuven, Belgium; 2 M3-BIORES: Measure, Model & Manage Bioresponses, Department of Biosystems, KU Leuven, Leuven, Belgium; 3 Department of Neurosurgery, University Hospitals Leuven, Leuven, Belgium

## Abstract

Conventional deep brain stimulation (DBS) applies constant electrical stimulation to specific brain regions to treat neurological disorders. Closed-loop DBS with real-time feedback is gaining attention in recent years, after proved more effective than conventional DBS in terms of pathological symptom control clinically. Here we demonstrate the conceptualization and validation of a closed-loop DBS system using open-source hardware. We used hippocampal theta oscillations as system input, and electrical stimulation in the mesencephalic reticular formation (mRt) as controller output. It is well documented that hippocampal theta oscillations are highly related to locomotion, while electrical stimulation in the mRt induces freezing. We used an Arduino open-source microcontroller between input and output sources. This allowed us to use hippocampal local field potentials (LFPs) to steer electrical stimulation in the mRt. Our results showed that closed-loop DBS significantly suppressed locomotion compared to no stimulation, and required on average only 56% of the stimulation used in open-loop DBS to reach similar effects. The main advantages of open-source hardware include wide selection and availability, high customizability, and affordability. Our open-source closed-loop DBS system is effective, and warrants further research using open-source hardware for closed-loop neuromodulation.

Deep brain stimulation (DBS) is a neurosurgical technique in which electrodes are implanted stereotactically in specific parts of the brain, and by applying electric currents, symptoms of various neurological disorders can be controlled. Because it is an invasive neurosurgical treatment with inherent surgical risk, it is mainly used to treat severe and otherwise-refractory diseases. Current clinical applications of DBS include movement disorders (e.g. Parkinson's disease), epilepsy, pain, and psychiatric disorders (e.g. obsessive-compulsive disorder and major depressive disorder).

The conventional DBS system is unidirectional: it delivers electrical stimulation without receiving any neural feedback. Recent technological breakthroughs make it possible to not only stimulate, but also record brain signals from relevant brain regions^1^,^2^. Based on neural inputs, stimulation can be adjusted in real-time, creating a closed-loop system. Closed-loop DBS has already proved to be more effective than conventional DBS in Parkinsonian symptom in animal research and clinical trials^1^,^2^. However, the availability of closed-loop DBS systems for research use is rather limited. An ideal closed-loop DBS system for research purposes is robust, reliable, affordable, and easily customizable. The recent emergence of open-source hardware introduced affordable and highly customizable hardware for various applications. Open-source closed-loop multichannel system for single-neuron manipulation has been investigated previously^3^. Here we describe conceptualization and validation of an open-source closed-loop DBS system for preclinical research purposes.

We used Arduino Uno (manufactured by Smart Projects, Italy), an open-source microcontroller, to control DBS system output (electrical stimulation) based on real-time input (neural signals). The input source is local field potentials (LFPs) from the hippocampus, and the output electrical stimulation is delivered in the mesencephalic reticular formation (mRt) in rats. Theta oscillations in the hippocampus are highly related to locomotion^4^, while electrical stimulation in the mRt induces freezing^5^. We hypothesize that our open-source closed-loop DBS system can suppress locomotion by stimulating the mRt based on real-time hippocampal theta power. To test our hypothesis, we measured the level of locomotion in rats under 4 different circumstances: no stimulation (OFF), open-loop stimulation (OL), random stimulation (RANDOM), and closed-loop stimulation (CL).

## Results

**Figure 1:**
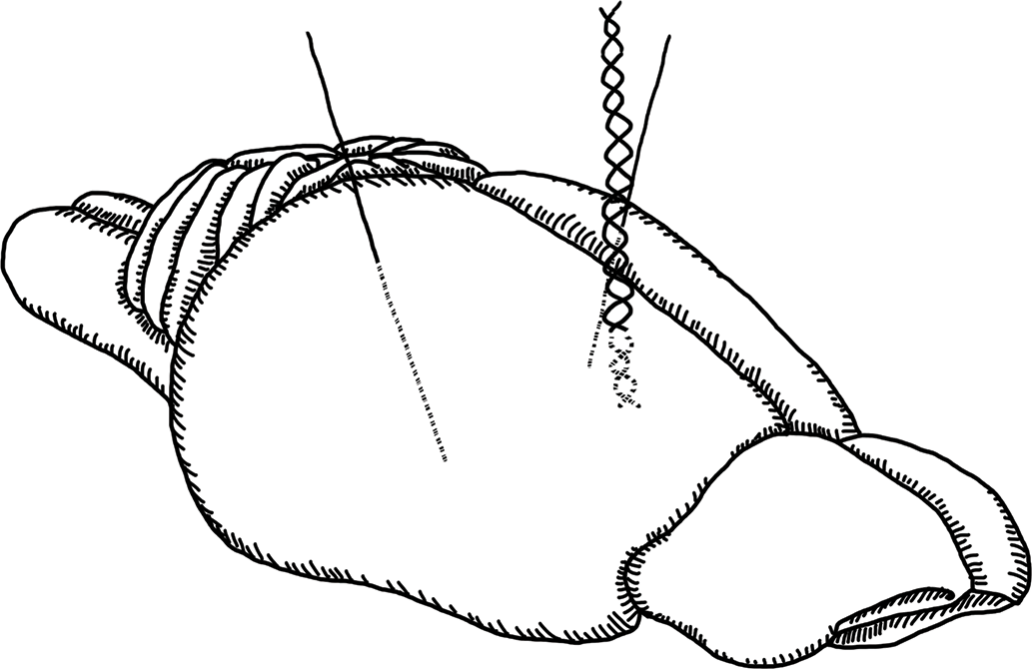
One bipolar and two monopolar electrodes were implanted in the right hippocampus (recording) and bilateral mesencephalic reticular formation (stimulation), respectively. Drawing by Stephany Pei-Yen Hsiao.

**Figure 2:**
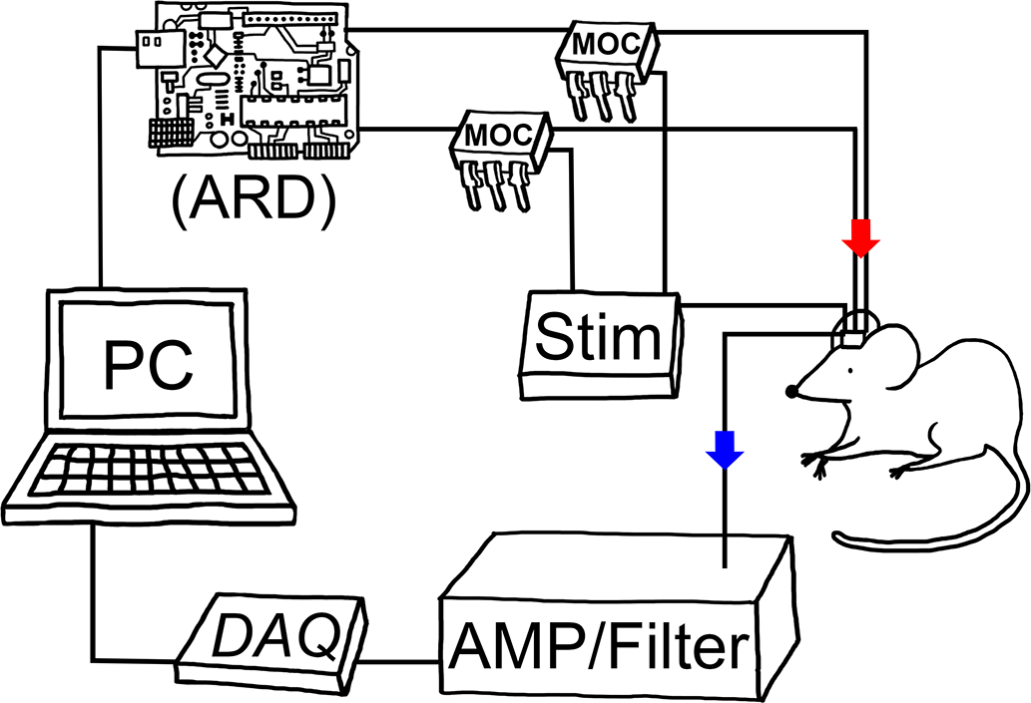
Schematic illustration of the open source, closed-loop deep brain stimulation system in rats. ↓(blue) indicates hippocampal local field potentials, recorded through amplifier, filter and data acquisition device, and analyzed in the PC. Based on real-time theta power analysis, electrical stimulation (indicated by ↓(red)) sent to the rat brain (mesencephalic reticular formation) is controlled via the Arduino board. AMP: custom amplifier, ARD: Arduino Uno board, DAQ: data acquisition card, MOC: mechanism operated cell, Stim: stimulator. Drawing by Stephany Pei-Yen Hsiao.

**Figure 3:**
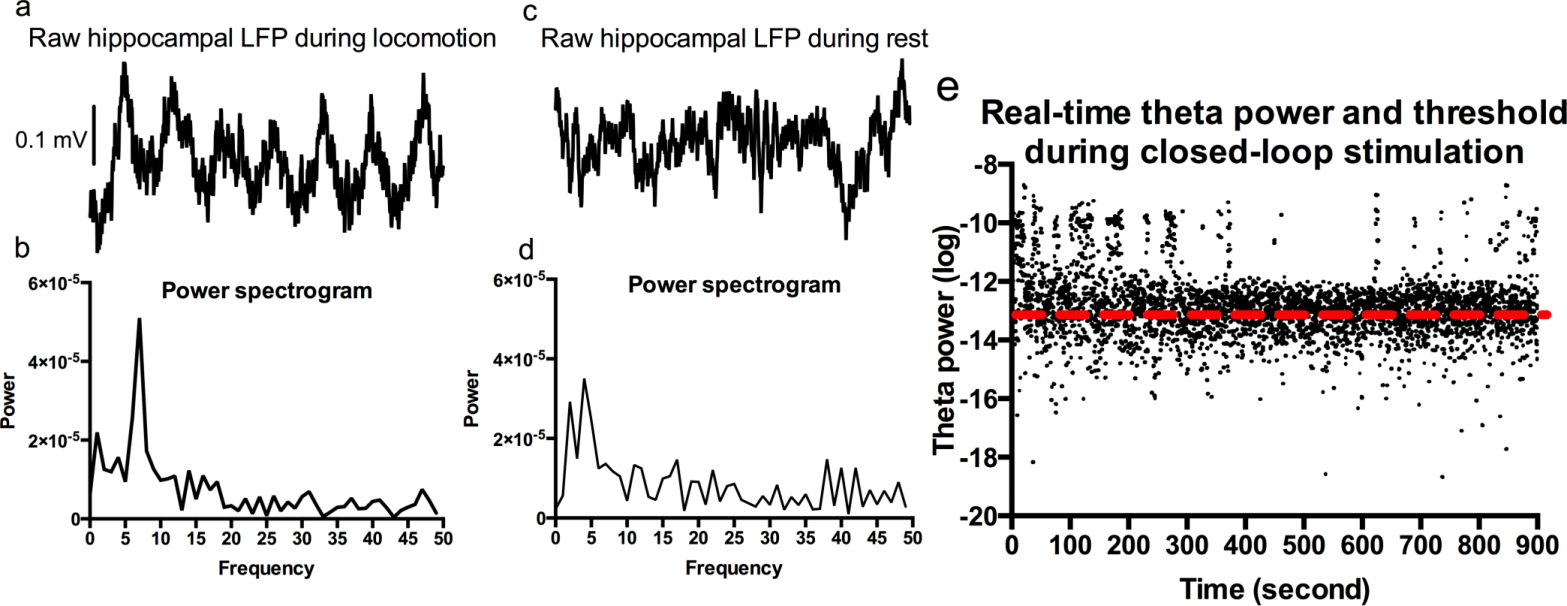
Measured hippocampal LFPs and theta power during closed-loop stimulation. *3a and b*: Rat hippocampal LFP and power spectrogram, showing a clear peak in theta band during locomotion. *3c and d*: Hippocampal LFP and power spectrums when the rat was resting. No peak in theta range was observed. *3e*: Real-time theta power during closed-loop stimulation. --- indicates the predetermined individual theta threshold. Each black dot represents real-time hippocampal theta power. If theta power exceeded the threshold (black dot above ---), bilateral stimulation in the mesencephalic reticular formation was switched on (until theta power dropped below threshold). LFP: local field potential.

The overview of hippocampal theta power threshold and mRt stimulation parameters of each individual rat is shown in Table 1. Figure 1 is the illustration of two monopolar and one bipolar electrodes implanted in the bilateral mRt and right hippocampus, respectively. In total 7 rats were included in the final analysis (5 dropouts: 4 misplaced electrodes, 1 premature death). The closed-loop hardware scheme is summarized in Figure 2. Figure 3 shows examples of hippocampal theta oscillations, and theta threshold during CL. The system delay time (from receiving input to actual output) was tested and estimated to be less than 100 milliseconds.

### Stimulation-on time in RANDOM and CL groups

The percentage of “stimulation-on time” during RANDOM and CL test sessions were 43.86 ± 0.80% and 55.57 ± 4.56%, respectively (mean ± standard error of the mean; paired t-test: p>0.05).

### Effects of Different Stimulation Schemes on Movement

The percentages of movement detected by automated video analysis during the 15-minute test sessions of OFF, OL, RANDOM, and CL were 62.40 ± 6.28%, 45.73 ± 5.38%, 67.80 ± 6.03%, and 44.60 ± 5.15%, respectively. Mauchly's test indicated that the assumption of sphericity had not been violated. RM-ANOVA showed that the effect of different interventions on percentage of movement was significant (p = 0.012). Post hoc pairwise comparisons (with Bonferroni corrections) indicated that the mean difference between OFF and CL was significant (p = 0.042).

**Figure 4:**
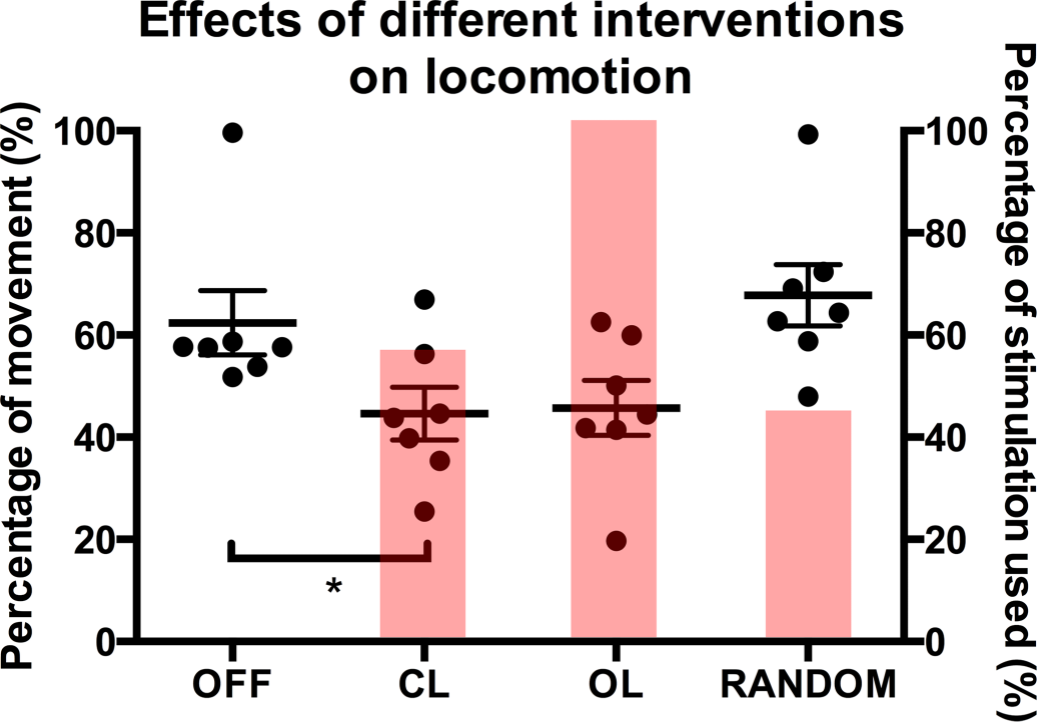
Effects of OFF, OL, RANDOM, and CL stimulations on locomotion (mean ± S.E.M., scatter plot), and corresponding percentage of stimulation-on time (mean, red columns). Repeated-measure analysis of variance showed that the main effect of different intervention on percentage of movement detected via automated video analysis was significant (p = 0.012). Post hoc pairwise comparisons (Bonferroni correction) indicated that the percentage of movement during CL was significantly lower than during OFF (p = 0.042). Percentages of stimulation-on time during RANDOM and CL were 43.86 ± 0.80% and 55.57 ± 4.56%, respectively. OFF: no stimulation, OL: open-loop stimulation, RANDOM: randomly-applied, CL: closed-loop. *: p<0.05.

Figure 4 summarizes the percentage of stimulation-on time and effect on movement during each stimulation scheme.

## Discussion

**Figure 5:**
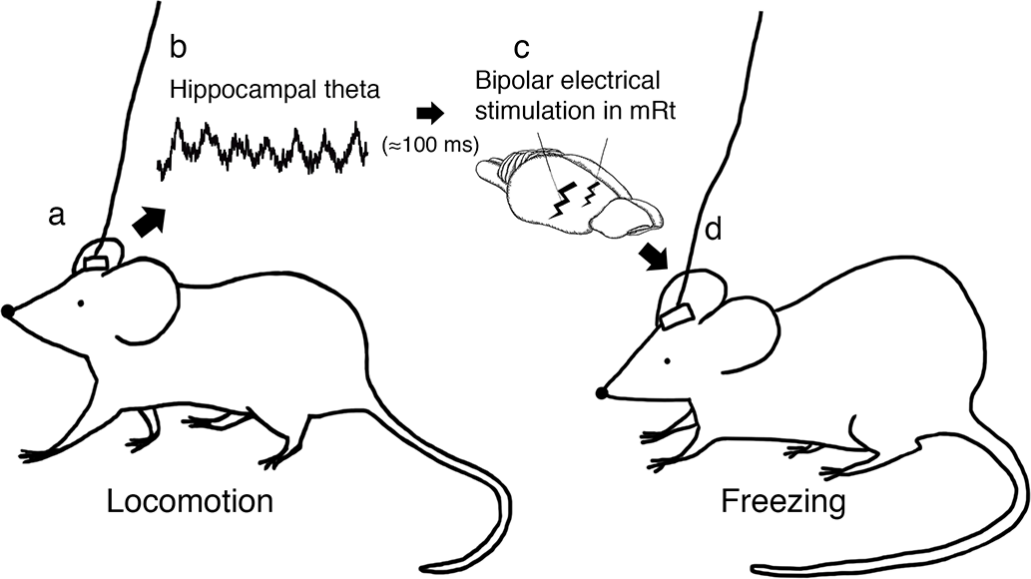
Graphical illustrations of hippocampal-mRt closed-loop deep brain stimulation. Locomotion (e.g. exploratory walking) in rat (*5a*) and corresponding hippocampal theta activity (*5b*, local field potential sample of 1 second), which triggers bipolar electrical stimulation in the mRt (*5c*), and induces freezing and suppresses locomotion (*5d*). Drawing by Stephany Pei-Yen Hsiao (5*a, c, and d*).

Our results showed that hippocampal-mRt closed-loop DBS significantly reduced locomotion compared to no stimulation. Open-loop mRt DBS also reduced locomotion compared to no stimulation (insignificantly in this study due to small sample size), in alignment with the results from previous study^5^. But with closed-loop DBS, only 55.57% of electrical stimulation was used compared to open-loop DBS to achieve similar effects. Electrical stimulation applied at random interval did not suppress locomotion, indicating that only electrical stimulation in the mRt given at the right moment can effectively suppress locomotion. Figure 5 summarizes the key steps of our closed-loop DBS.

A dynamic system is a system whose behavior changes over time, mostly in response to external stimulation/disturbances. A closed-loop system then, refers to a situation in which two or more dynamic systems are interconnected to each other in a cycle, such that each system influences the other and the dynamics of each system are strongly coupled. When there are two systems for instance, the first system influences the second system which in turn influences the first system by giving feedback, this feedback from the second to the first system makes the whole system a closed-loop^6^. Based on the measured output compared to a set of reference values, the error on the system output is measured. When this error reaches a predefined threshold value, the system input is changed by a controller in order to adapt the system output, hence decreasing the error on the output to an acceptable value^7^.

The advantage of a closed-loop control system lies in the fact that feedback control algorithms are designed to acquire the desired performance by altering the inputs immediately once deviations are observed regardless of what caused the disturbance^8^. In the case of closed-loop neuromodulation, the central nervous system acts as controller of many body systems at organism scale (e.g. control of movement), and control of the central nervous system by DBS is a promising example of how control theory can be applied to adapt (pathological) behavior of organisms.

In our study, we have shown that closed-loop DBS is effective in suppressing locomotion with less electrical stimulation used compared to open-loop DBS. This implicates the advantages of less stimulation-induced side effects and more efficient use of energy of closed-loop DBS during clinical application. To the best of our knowledge, this is the first attempt to use hippocampal-LFP-based neuromodulation to manipulate behavior of rodents. In principle, we've proven that open-source hardware is capable of effectively intervening neural circuits in a closed-loop fashion. The system delay time (from receiving input to actual output) of less than 100 milliseconds seemed acceptable in our model, and was comparable to other closed-loop neural stimulation systems^1^,^2^. The main component of the delay came from the processing of the LFP data in Matlab. We also have delay from the Arduino microcontroller (in the order of µsec) and the mechanism operated cell (also in the order of µsec) for on- and off-switching. The DAQ card is another source of delay, in the range of a few milliseconds maximally. Optimization of hardware (with more powerful chipsets) and software setup (enhanced algorithms) may further reduce delay and improve system efficacy. With our current setup, the open source hardware component is only acting as a controller of output based on input. This is related to limitations of Arduino Uno, but with more advanced open source hardware systems (e.g. open source mini pc and data acquisition system), it is possible to build a complete open source closed-loop neural stimulation system.

The main advantages of open source hardware include wide range of selection and availability, high customizability, and affordability. Our results suggest open source hardware as an effective component for closed-loop DBS system, and warrant future research of closed-loop neural stimulation using open source hardware.

## Methods

### Study overview

12 male Wistar rats weighing 200–250 g were used in our study. One twisted bipolar electrode and 2 monopolar electrodes were implanted in the hippocampus and the mRt in each rat, respectively. After one week of recovery, all rats underwent 1 day of baseline measurement followed by 4 days of testing sessions. The level of locomotion of every rat during each testing session was analyzed and compared statistically (see text below for more details).

This research project and the experimental protocol were approved by the KU Leuven ethics committee for laboratory experimentation (project number: P093/2012), and was in accordance with the Belgian and European laws, guidelines and policies for animal experimentation, housing and care (Belgian Royal Decree of 29 May 2013 and European Directive 2010/63/EU on the protection of animals use for scientific purposes of 20 October 2010).

### Surgical procedures

Rat was anesthetized (Anesketin (0.06 mL/100 g body weight) and Domitor (0.04 mL/100 g body weight)), put on a heating pad with anal probe to keep the body temperature at approximately 37.5°C, and properly fixed in a stereotactic frame. Midline incision and three burr holes were made based on implantation trajectory (1 for hippocampus and 2 for mRt; coordinates of mRt: 5.76 mm posterior to bregma, 3.4 mm lateral to midline, 6 mm deep relative to dura, 14° to sagittal insertion angle; coordinates of hippocampus: 4 mm posterior to bregma, 2.8 mm lateral to midline, 2.7 mm deep relative to dura, 20° to sagittal insertion angle). Three surrounding burr holes were made (1 for reference screw (E363/20, PlasticsOne), 2 for anchoring screws) before 2 monopolar electrodes (E363/8, PlasticsOne) and 1 bipolar electrode (E363/8-2TW, PlasticsOne) were implanted in the mRt and the hippocampus, respectively. After mounting reference screw and anchoring screws, dental cement was applied and a plastic pedestal (MS363, PlasticsOne) was fixed on top of rat's head, with sockets of implanted electrodes placed inside. Antisedan (0.03 mL/100 g body weight) was administered after operation was completed, and each rat was given one week of recovery.

### Experimental setup

The test cage was 34×34×34 cm. Each rat was placed individually in the test cage for 15 minutes^9^,^10^,^11^ each day during baseline and testing sessions. When rat was placed in test cage, the pedestal was connected to a swivel (swivel: SL6C, PlasticsOne; wire: 363-363 (CS), PlasticsOne). LFP was recorded in every rat during baseline and test sessions via a custom filter device^12^ to filter out stimulation artifact, a preamplifier (66 dB) to increase signal-to-noise ratio, and a data acquisition card (NI USB-6341, National Instruments, Texas, USA; software environment: MatLab, MathWorks, Natick, MA, USA). Hippocampal LFPs were recorded at 10 kHz. To extract relevant information, two filters were applied: one Butterworth bandpass (1–300 Hz) and one notch (49–51 Hz). An Arduino Uno board was connected between processed input and stimulator output, to steer stimulation based on hippocampal LFPs. A webcam (Logitech HD Webcam Pro C910) was fixed on top of the cage to record behavior of rat. In total 15 hours of LFPs and videos were recorded.

After one week of recovery from surgery, each rat underwent 1 day of baseline measurement and 4 days of testing period. During baseline measurement, hippocampal theta threshold and optimal stimulation parameters were determined. During the 4-day testing period, each rat underwent one of the 4 following intervention each day (random, non-repetitive): no stimulation (OFF), open-loop stimulation (OL), random stimulation (RANDOM) and closed-loop stimulation (CL). During OFF, no electrical stimulation was applied in the mRt; during OL, stimulation was constantly applied in the mRt; during RANDOM, certain percentage (determined during baseline measurement, see text below for details) of “stimulation-on time” was applied randomly; during CL, stimulation was applied only when real-time hippocampal theta power exceeded the threshold.

The percentage of movement during each 15-minute test session of each rat was then evaluated by automated video analysis (see text below for more details). We used cresyl violet staining method to examine the three implanted locations (one in right hippocampus, and two in left and right mRt). Rats with misplaced electrodes were excluded from statistical analysis.

### Automated video analysis

To perform automated video analysis, an algorithm to detect movement in a video recording of a rat was developed. The two main measurements of automated video analysis were: percentage of movement, and the exact time of movement. The algorithm was based upon a Matlab script developed by Tambuyzer et al. to measure travelled distance in an experiment with rats on compulsive behavior^13^. This algorithm was used for both baseline measurements and for movement analysis on the video recordings during testing period. In brief, the major steps of video analysis are as follows: each frame of all 15-minute videos (15 frames per second) was first converted to black & white image, and the border of the test cage was automatically detected. A specific grey-scale value was used to separate the rat (white) from background (dark), and the centroid point of the rat was obtained for each frame. A binary value representing movement (yes/no) between frames was then calculated. Definition of movement: when the total change in centroid position over 3 consecutive frames exceeded 5 pixels (approximately 0.5 cm), the rat was considered to be moving. Lastly, percentage of movement (percentage of time during the recording that the rat spent moving), and the exact time of movement were obtained.

### Baseline measurement

Two main goals were achieved during baseline measurement: calculation of hippocampal theta power threshold and optimization of mRt stimulation parameters.

The hippocampal theta power threshold was obtained during baseline measurement, and served as a real-time neurophysiological indicator of locomotion. The rat was placed in the test cage for 15 minutes during baseline measurement. Hippocampal LFP and behavior (video) were measured and analyzed offline. Percentage of movement and exact moments of movements (time points) were extracted from behavioral measurement based on automated video analysis. Medians and standard deviations of power spectral density at specific theta frequencies (7.8 Hz and 9.8 Hz) were obtained from LFPs (window size: 500 ms, 250 ms overlap). The hippocampal theta power threshold (*Threshold*) was defined in the following equation: 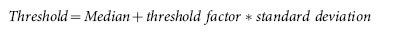


A set of threshold factors (0.1–0.5 in 0.05 increment) was tested in both sets of median and standard deviation against behavioral data, and the combination of frequency and threshold factor with the highest accuracy to predict movement was chosen as the hippocampal theta power threshold for a rat. The time delay between the exceeding of threshold value in the LFP data and the actual start of movement seen in the video and vice versa was also taken into account, e.g. if the theta power exceeded threshold value 200 ms before the movement was detected on video, this was still considered a true positive (maximum allowed time delay: 250 ms). Stimulation was switched on for 500 ms once the real-time theta power exceeded Threshold.

Optimization of mRt stimulation parameters was done after LFP and behavioral measurements. Electrical stimulations with different frequencies, pulsewidths, and amplitudes were tested in each rat to achieve maximal freezing without observable side effects (e.g. epileptic behaviors).

After baseline measurements, a set of hippocampal theta power threshold and mRt stimulation parameters in each individual rat was obtained, and would be used in the following testing period.

Besides hippocampal theta power threshold and optimal stimulation parameters, the percentage time of locomotion would be used as the percentage of “stimulation on” time during RANDOM testing, with stimulation applied in random time points without regards to the rat's behavioral state.

### Statistical analysis

The percentages of “stimulation-on time” during RANDOM and CL were compared to examine the level of significance of the difference of sample means (paired t-test). One-way repeated measures analysis of variance (RM-ANOVA) was used to examine if the main effect of different intervention between groups on percentage of locomotion was significant. We used Statistica (StatSoft Inc., Tulsa, OK, USA) to perform statistical analysis (the level of significance was set at 0.05 for all statistical tests).

## Author Contributions

HW, HG, and DB drafted the manuscript and designed the experiment, HG and TT set up the hardware system, DB performed the experiment and collected the data, HW, KvK and TT analyzed the data, HW, HG, and TT contributed to statistical analysis, and HW, KvK, JMA and BN finalized the submitted manuscript. All contributors critically reviewed and approved the manuscript.

## Figures and Tables

**Table 1:** Theta threshold values (logarithmic) and corresponding theta frequencies, and stimulation parameters (amplitudes, bandwidths, and frequencies) of each rat during test sessions.

	Theta threshold	Stimulation parameters
Rat 1	–13.61@7.8 Hz	300 uA, 60 us, 130 Hz
Rat 2	–13.20@9.8 Hz	220 uA, 50 us, 130 Hz
Rat 3	–13.19@9.8 Hz	400 uA, 60 us, 130 Hz
Rat 4	–13.19@9.8 Hz	210 uA, 50 us, 130 Hz
Rat 5	–13.70@7.8 Hz	210 uA, 60 us, 130 Hz
Rat 6	–13.64@7.8 Hz	200 uA, 60 us, 130 Hz
Rat 7	–13.30@7.8 Hz	160 uA, 50 us, 130 Hz
