# Erratum: Conceptualization and validation of an open-source closed-loop deep brain stimulation system in rat

**DOI:** 10.1038/srep46954

**Published:** 2018-03-19

**Authors:** Hemmings Wu, Hartwin Ghekiere, Dorien Beeckmans, Tim Tambuyzer, Kris van Kuyck, Jean-Marie Aerts, Bart Nuttin

Correction to: Scientific Reports 5: Article number: 992110.1038/srep09921; published online: 04 21 2015; updated: 03 19 2018

The original version of this Article contained a typographical error in the volume number ‘5’ was incorrectly given as ‘4’. This error has now been corrected in the PDF and HTML versions of the Article.

